# Beyond HPV16/18: genotype distribution of high-risk HPV and cycle threshold values in cervical carcinogenesis spectrum

**DOI:** 10.1080/07853890.2026.2746866

**Published:** 2026-10-15

**Authors:** Esra Isci Bostanci, Suheyl Korhan, Selin Yigit, Elif Caliskan, Gulendam Bozdayi

**Affiliations:** ^a^ Faculty of Medicine, Obstetrics and Gynecology, Division of Gynecologic Oncology, Gazi University, Ankara, Turkey; ^b^ Faculty of Medicine, Medical Microbiology, Division of Virology, Gazi University, Ankara, Turkey

**Keywords:** Non-16/18 HR-HPV genotypes, Ct values, cervical carcinogenesis

## Abstract

**Background:**

Cervical cancer is a virus-dependent gynaecologic malignancy and according to GLOBOCAN 2022 cervical cancer is the fourth frequent women cancer in all over the world. Despite this frequency it is rarely seen in developed countries because of the enforcement of cervical screening programmes with human papillomavirus (HPV) test and Pap test called cotest. The burden of this women cancer is high despite the implementation of HPV-based vaccines and screening strategies, could nearly eliminate cervical cancer as a public health problem.

**Materials and Methods:**

A retrospective analysis was conducted using non-16/18 high risk human papilloma virus (HR-HPV) genotyping data and cycle threshold (ct) values from women aged ≥18 years that underwent cervical sampling during gynaecological outpatient clinic at a tertiary centre in Turkey, between January 2022 and December 2023.

**Results:**

The most frequently detected genotypes were HPV52 (*n* = 24), HPV31 (*n* = 23), HPV51 (*n* = 22) and HPV56 (*n* = 22). On the other hand in cervical intraepithelial neoplasia 3 (CIN3) cases, with combinations involving HPV51, HPV52, HPV59, HPV66 and HPV31 commonly detected. Median minimum patient-level Ct values were 22.0 (Interquartile range-IQR 8) in chronic cervicitis, 19.0 (IQR 10) in CIN1, 17.0 (IQR 8) in CIN2 and 19.0 (IQR 7) in CIN3.

**Conclusion:**

To the best of our knowledge, there are limited data about cycle threshold values in prediction of viral load. These region-specific, genotype-resolved data may inform extended-genotyping–based risk stratification and support ongoing surveillance of non-16/18 HR-HPV patterns within HPV-based screening programs. Hence this study opens a new perspective in this era.

## Introduction

Despite being largely preventable through evidence-based screening and prophylactic vaccination, cervical cancer remains a major global health burden. In 2020, an estimated 604,000 new cases of cervical cancer and 342,000 related deaths occurred worldwide. There is a disproportionate mortality rate in low- and middle-income settings [1]. In response, the global elimination of cervical cancer as a public health problem has been proposed as an achievable target through the accelerated implementation of HPV vaccination, HPV-based screening and timely treatment of precursor lesions [2].

Persistent infection with high-risk human papillomavirus (HR-HPV) is necessary for the development of cervical intraepithelial neoplasia (CIN) and invasive cervical carcinoma [3,4]. Although HPV16 and HPV18 are responsible for the majority of cervical cancer cases worldwide, mounting evidence suggests that a significant proportion of high-grade lesions and cancers are caused by non-16/18 HR-HPV genotypes, which vary in distribution and oncogenic potential across populations. In the post-vaccination era, increasing attention has been directed towards potential shifts in HR-HPV genotype prevalence, underscoring the need for ongoing surveillance of type distribution [5,6].

Beyond HPV16/18, several genotypes – including HPV31, HPV33, HPV45, HPV52 and HPV58 – have been consistently associated with CIN2/3 and invasive cervical cancer, although their genotype-specific risks and relative contributions vary across geographic regions [6,7]. This geographic heterogeneity is clinically significant because the risks of persistence and progression specific to a given genotype directly affect the probability of developing CIN2+ lesions and cervical cancer [8]. Host and population factors (age, immune status, screening coverage, vaccine uptake) shape genotype distribution. These factors may also influence persistence and progression dynamics [9,10]. Therefore, it is essential to define the local distribution of non-16/18 HR-HPV genotypes and their association with lesion severity in order to refine prevention strategies and improve individualized risk stratification [6,7].

The cycle threshold (CT) value obtained in Polymerase Chain Reaction (PCR)-based HPV tests is a semi-quantitative metric reflecting the amplification cycle at which the target nucleic acid signal reaches a detectable level, and this value is considered an indirect indicator of viral load [11].

Current risk-based cervical screening and management algorithms emphasize HPV16/18 positivity because these genotypes pose the greatest immediate risk, often justifying expedited colposcopy or treatment [12]. However, emerging evidence from extended genotyping-based risk stratification suggests that several non-16/18 HR-HPV genotypes pose a clinically significant risk of CIN2+. This indicates that triage strategies that focus primarily on HPV16/18 may overlook a subset of women who are at an increased risk [7]. Moreover, multiple HR-HPV infections further complicate risk estimation, and the clinical implications of genotype combinations remain incompletely characterized. Type-specific natural history data indicate that risk trajectories differ substantially across HRHPV genotypes, whereas the incremental prognostic value of multiple-type infections remains uncertain in routine screening populations. These considerations underscore the need for region-specific, real-world evidence that distinguishes individual HR-HPV genotypes and evaluates their associations with cervical disease severity [10].

Screening is carried out every three years with a Pap-cytology test for women aged 21–30, and every five years with co-testing (HPV and cytology) for women aged 30–65, within the scope of the national cervical cancer screening programme in Turkey. Although a well-settled and effectively implemented HPV screening program exists in our country, HPV vaccination is not included in the national immunization program. Strengthening local data is essential to support genotype-informed risk stratification, refine triage pathways within HPV-based screening programs, and inform vaccine policy, considering the potential benefits of broader-valency vaccines. Accordingly, this study aimed to characterize the distribution of non-16/18 HR-HPV genotypes and their associations with histopathologic severity (chronic cervicitis to CIN3) among women undergoing histopathologic evaluation in an Ankara cohort.

## Materials and methods

### Study design and setting

This retrospective, single-centre study was conducted at a tertiary care gynaecology outpatient clinic in Ankara, Türkiye. Women aged ≥18 years who underwent cervical sampling as part of routine clinical care between January 2022 and December 2023 were evaluated.

### Study population and eligibility criteria

During the study period, 5,446 cervical samples submitted for HPV testing had available high-risk HPV (HR-HPV) results and subsequent genotyping data. Patients were excluded if cervical samples were inadequate for HPV testing, genotyping results were missing, or relevant clinical/histopathologic data were incomplete.

### HPV testing and extended genotyping

Cervical specimens were collected during routine gynaecologic outpatient visits and processed according to standard laboratory procedures. Primary HR-HPV screening was performed using a real-time PCR assay (cobas^®^ 4800 HPV Test; Roche Diagnostics, Switzerland), with HPV16 and HPV18 reported separately and all other high-risk types classified as non-16/18 hrHPV. HrHPV-positive samples subsequently underwent extended genotyping using the NLM Genotypes 14 Real-TM Quant assay (NLM Diagnostic, Italy) on the Rotor-Gene Q platform (QIAGEN, Germany), enabling genotype-resolved detection of HPV31, HPV33, HPV35, HPV39, HPV45, HPV51, HPV52, HPV56, HPV58, HPV59, HPV66 and HPV68.

### Cycle threshold (Ct) values

Cycle threshold (Ct) values were extracted for each detected genotype and interpreted as a proxy for viral burden, with lower Ct values reflecting higher viral load. Ct values were recorded separately for each detected hrHPV genotype from the extended genotyping assay. In multiple-type infections, Ct values were recorded per genotype, and the minimum Ct value per individual was used for patient-level analyses to summarize the strongest viral-burden signal. For genotype-specific descriptive analyses, Ct values were categorized as low (0–<10), intermediate (10–<20) and high (≥20).

### Histopathology and referral cohort

Histopathologic outcomes were retrieved from pathology reports of clinically indicated cervical biopsy and/or excisional specimens and categorized as chronic cervicitis, CIN1, CIN2, or CIN3. When multiple specimens were available, the highest-grade diagnosis was retained. Analyses linking HPV genotypes and Ct values to lesion severity were restricted to women with available histopathologic evaluation.

### Outcomes

The primary outcome was the distribution of non-16/18 HR-HPV genotypes. Secondary outcomes included genotype-specific associations with histopathologic severity (chronic cervicitis, CIN1, CIN2 and CIN3), comparisons of genotype-level and patient-level Ct values across histopathologic categories, and evaluation of single-type versus multiple-type HR-HPV infections in relation to lesion severity. High-grade disease was defined as CIN2+ (CIN2 or CIN3).

### Statistical analysis

Descriptive statistics were used to summarize demographic characteristics, genotype distributions, infection patterns, histopathologic outcomes and Ct values. Continuous variables are presented as mean ± standard deviation (SD) or median and interquartile range (IQR), as appropriate and categorical variables had given as number and percentage. Given the small number of cases in some histopathologic categories, particularly CIN2 and the resulting sparse cell counts and multivariable regression analyses were not performed. All descriptive analysis were conducted using IBM SPSS Statistics (version 25; IBM Corp., Armonk, NY, USA).

### Ethics

The study protocol was approved by the Gazi University Ethics Committee (approval no: 2025-1558, date: 22.09.2025). The study was conducted in accordance with the Declaration of Helsinki. Given the retrospective design and use of de-identified clinical data, the requirement for informed consent was waived in accordance with institutional policy and national regulations.

## Results

### Study population and histopathologic outcomes

Among 5,446 cervical samples evaluated during the study period, 121 women underwent clinically indicated biopsy and had available histopathologic evaluation; these women constituted the referral cohort. The 121 women indeed represent a referral population who underwent biopsy due to abnormal cytology (ASC-US or worse) or other specific clinical indications following a positive non-16/18 HPV test. The mean age was 43.36 ± 10.15 years (range: 24–73 years). Histopathologic examination revealed chronic cervicitis in 48.8% (*n* = 59), CIN1 in 28.9% (*n* = 35), CIN2 in 3.3% (*n* = 4) and CIN3 in 19.0% (*n* = 23). The demographic and clinical characteristics of the study population are summarized in Table 1.

**Table 1.** Demographic and clinical characteristics of the study population (*n* = 121).

Variable	Value
Age, years	43.36 ± 10.15 (24–73)
Histopathology	
Chronic cervicitis	59 (48.8%)
CIN1	35 (28.9%)
CIN2	4 (3.3%)
CIN3	23 (19.0%)
Infection pattern	
Single-type hrHPV	69 (57.0%)
Multiple-type hrHPV	52 (43.0%)

Values are presented as mean ± standard deviation (range) or *n* (%).

### Infection patterns

Single-type HR-HPV infection was identified in 57.0% (*n* = 69), whereas multiple-type infections were observed in 43.0% (*n* = 52).

### Distribution of non-16/18 high-risk HPV genotypes

A total of 197 non-16/18 HR-HPV genotype detections were recorded across the analytic cohort. Because multiple infections were common, counts represent total genotype detections rather than unique patients. The most frequently detected genotypes were HPV52 (*n* = 24), HPV31 (*n* = 23), HPV51 (*n* = 22) and HPV56 (*n* = 22). Other commonly detected genotypes included HPV66 (*n* = 19), HPV59 (*n* = 16), HPV39 (*n* = 15), HPV68 (*n* = 15), HPV33 (*n* = 14), HPV58 (*n* = 13), HPV45 (*n* = 8) and HPV35 (*n* = 6). The distribution of non-16/18 HR-HPV genotype detections is summarized in Table 2 and Figure 1.

**Figure 1.**
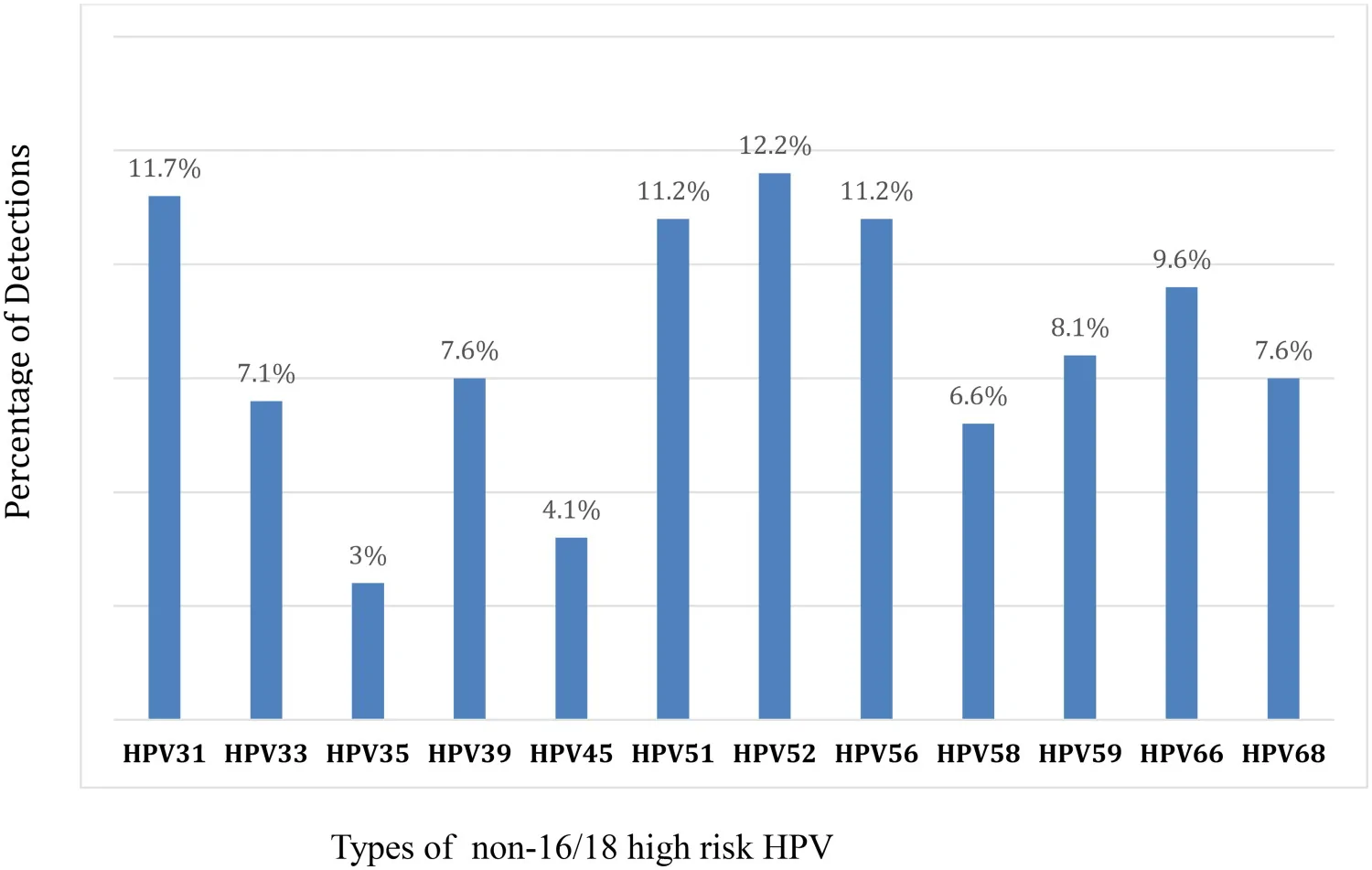
Distribution of non-16/18 high risk HPV types. Types of non-16/18 high risk HPV

**Table 2.** Distribution of non-16/18 high-risk HPV genotype detections (*n* = 197).

Genotype	Detections, *n*	% of detections*
HPV52	24	12.2
HPV31	23	11.7
HPV51	22	11.2
HPV56	22	11.2
HPV66	19	9.6
HPV59	16	8.1
HPV39	15	7.6
HPV68	15	7.6
HPV33	14	7.1
HPV58	13	6.6
HPV45	8	4.1
HPV35	6	3.0
**Total**	**197**	**100.0**

* Counts represent total genotype detections rather than unique patients because multiple-type infections were common.

### Genotype distribution in CIN2+ and CIN3 cases

Overall, CIN2+ lesions were identified in 22.3% (*n* = 27), including CIN2 in 3.3% (*n* = 4) and CIN3 in 19.0% (*n* = 23). Within CIN2+ cases, a heterogeneous distribution of non-16/18 HR-HPV genotypes was observed, most frequently including HPV52, HPV51, HPV31 and HPV56, with additional detections of HPV33, HPV59, HPV66, HPV58, HPV45, HPV39 and HPV35.

Among CIN3 cases (*n* = 23), both single-type and multiple-type HR-HPV infections were identified. Multiple-type infections were observed in a substantial proportion of CIN3 cases, with combinations involving HPV51, HPV52, HPV59, HPV66 and HPV31 commonly detected. Single-type infections were also present and most frequently involved HPV51, HPV52, HPV31, HPV33 and HPV56. The distribution of non-16/18 HR-HPV genotype detections in CIN2+ and CIN3 lesions is summarized in Table 3.

**Table 3.** Distribution of non-16/18 high-risk HPV genotype detections in CIN2+ and CIN3 lesions.

Genotype	CIN2+ (*n*)	CIN3 (*n*)
HPV31	7	3
HPV33	4	4
HPV35	1	1
HPV39	1	1
HPV45	2	1
HPV51	8	7
HPV52	8	8
HPV56	5	3
HPV58	1	1
HPV59	3	3
HPV66	4	3
HPV68	2	2

Counts represent total genotype detections rather than unique patients. CIN2+ includes CIN2 and CIN3.

### Ct value analysis (minimum patient-level Ct)

Median minimum patient-level Ct values were 22.0 (IQR 8) in chronic cervicitis, 19.0 (IQR 10) in CIN1, 17.0 (IQR 8) in CIN2 and 19.0 (IQR 7) in CIN3. The observed minimum Ct values ranged from 9 to 32 in chronic cervicitis, 8 to 32 in CIN1, 10 to 20 in CIN2 and 11 to 31 in CIN3. Patient-level minimum Ct values across histopathologic categories are summarized in Supplementary Table S1. On the other hand HPV genotype-specific Ct analyses mentioned in Supplementary Table S3.

Given the small number of CIN2 cases and sparse genotype-specific cell counts, formal statistical comparisons across histopathologic categories were not performed, and results are presented descriptively.

### Histopathological distribution of Ct categories

Histopathological distributions of Ct categories (low, intermediate, high; 0–<10, 10–<20, ≥20) across histopathologic outcomes are presented in Supplementary Table S1. Due to small genotype-specific cell counts, these analyses are descriptive. The distribution of infection patterns according to histopathologic category is indicated in Supplementary Table S2.

## Discussion

This study provides a detailed characterization of non-16/18 high-risk HPV genotype detections across histopathologic outcomes, with particular emphasis on genotype distribution, infection patterns and patient-level Ct metrics.

In this histopathology-evaluated cohort, chronic cervicitis was the most frequent diagnosis, while CIN2+ lesions were identified in 22.3% of women. This spectrum is compatible with referral-based practice, where the yield of high-grade disease depends on the referral pathway and screening context; in settings transitioning to primary HR-HPV screening, increased colposcopy referrals and detection of low-grade findings have been reported [13]. Consistent with our case-mix, a recent HR-HPV positive referral cohort with normal or minor cytologic abnormalities demonstrated CIN2+ detection rates in a comparable range, varying by cytology category [14]. The mean age in the early forties also aligns with evidence that non-16/18 genotype patterns in high-grade disease show age-related shifts, with a stronger contribution of HPV16/18 at younger ages and a relatively greater contribution of non-16/18 genotypes at older ages [15].

In our cohort, multiple-type HR-HPV infections were common among women undergoing histopathologic evaluation, a finding consistent with contemporary referral-based series reporting frequent coinfections in histopathologically confirmed cervical lesions [16]. In large clinical datasets, multiple HR-HPV infections have generally not been associated with a cumulatively higher risk of CIN2+ compared with the corresponding single-genotype infections, supporting the concept that coinfections do not necessarily imply synergistic oncogenic progression [17]. Similarly, a recent study reported that multiple HR-HPV infections were associated with a higher risk of low-grade cytologic abnormalities but not with high-grade intraepithelial lesions, reinforcing the need for cautious interpretation of multiple-type detections in clinical triage [18]. Accordingly, in studies where multiple genotypes are detected in high-grade lesions, genotype detections should be interpreted primarily as co-occurrence at the patient level rather than direct etiologic attribution to each detected genotype [19].

In this retrospective cohort, HPV52 and HPV31 were the most frequently detected non-16/18 high-risk HPV genotypes, followed by HPV51 and HPV56.

These findings are consistent with the most recent global causal attribution analysis showing that, after HPV16/18, HPV31/33/45/52/58 together contribute an additional 15–20% of invasive cervical cancer cases worldwide [20].

The same analysis reports regional variation in genotype-attributable fractions (e.g. a higher contribution of HPV35 in Africa), supporting that genotype patterns differ across settings and populations [21]. In our cohort, non-16/18 genotypes – most commonly HPV52, HPV51, HPV31 and HPV56 – were frequently detected among women with CIN2+ and CIN3.

In age-stratified CIN3 data, the proportion of CIN3 associated with HPV16/18 decreases with increasing age, while non-16/18 genotypes become relatively more prevalent among older women. In histopathology-confirmed cervical squamous lesions, multiple HR-HPV infections are frequently observed, and large tertiary-care data suggest that multiplicity does not necessarily confer higher CIN3+ detection compared with corresponding single-type infections, with some genotype combinations showing lower CIN3+ detection rates [22,23].

At the population level, a recent systematic review and meta-analysis emphasized that regional differences in single versus multiple HPV infection patterns have not been consistently characterized and reported substantial heterogeneity across regions, supporting cautious interpretation of multiplicity across cohorts [20].

In this study, median minimum patient-level Ct values decreased from chronic cervicitis to CIN2 and showed partial overlap across CIN categories, consistent with prior evidence supporting an inverse relationship between Ct (viral load proxy) and histologic severity at the population level [21]. Nevertheless, Ct distributions across grades are known to overlap substantially, and group-level trends do not necessarily translate into individual-level discrimination [22]. The slightly higher median Ct observed in CIN3 than CIN2 in our dataset is plausibly explained by the small CIN2 sample size and measurement variability, as Ct metrics are influenced by assay characteristics and sampling factors [24]. Accordingly, genotype-specific Ct category analyses were presented descriptively, aligning with prior extended-genotyping work cautioning against inferential genotype-level Ct comparisons in small high-grade strata [7]. Overall, our findings support interpreting Ct as an adjunctive, context-dependent marker rather than a standalone triage tool in routine practice [12].

This study has several limitations. First, its retrospective, single-centre design may limit generalizability and introduces referral bias, as histopathologic evaluation was available only for women undergoing clinically indicated biopsy. Second, the small number of CIN2 cases constrained statistical inference and reduced the ability to assess dose–response patterns in Ct values across histologic grades. Third, the high frequency of multiple-type hrHPV infections limited genotype-specific attribution and reduced the power of genotype-level Ct comparisons. Fourth, Ct values represent an indirect proxy of viral burden and are influenced by sampling adequacy and assay-specific characteristics; therefore, Ct-based findings may not be directly transferable to other platforms or settings. Finally, genotype-specific Ct category analyses were necessarily descriptive due to small cell counts, and should be interpreted as hypothesis-generating. Another limitation was the unknown vaccination status of a portion of the cohort. Despite these; this study provides a hypothesis in the need of the other types of HPV like 51, 56, 59, 66 in vaccination. Therefore larger studies needed to be done to make this prediction.

## Conclusion

Our findings demonstrate that non-16/18 HR-HPV genotypes constitute a heterogeneous component of cervical pathology in an Ankara referral cohort, with HPV52, HPV31, HPV51 and HPV56 among the most frequently detected types. A potential area of interest could be the association of CIN2+ cases with HPV51, HPV56, HPV59 and HPV66, given that they are not included in existing prophylactic vaccines. In this direction, our aim is to point out the need for further research into the potential role of these genotypes in cervical carcinogenesis and their possible consideration in future vaccine formulations. Although minimum patient-level Ct values showed a general tendency towards lower values in higher-grade lesions, substantial overlap across histologic categories supports interpreting Ct as an adjunctive, context-dependent marker rather than a standalone triage metric. These region-specific, genotype-resolved data may inform extended-genotyping–based risk stratification and support ongoing surveillance of non-16/18 HR-HPV patterns within HPV-based screening programs.

## Supplementary Material

Supplementary Table S1.docx

Supplementary Table S2.docx

Supplementary Table S3.docx

## Data Availability

The data presented in this study are available on reasonable request from the corresponding author.
