# Supplementary material for: Beyond HPV16/18: genotype distribution of high-risk HPV and cycle threshold values in cervical carcinogenesis spectrum
Source: Ann Med. 2026 Oct 15;58(1):2746866. doi: 10.1080/07853890.2026.2746866 (PMC13674648; doi:10.1080/07853890.2026.2746866)
Supplement: Supplementary file 1 [file IANN_A_2746866_SM6828.docx]

**Supplementary Table S3.** Genotype-specific cycle threshold (Ct) values

| **HPV genotype** | **Ct, median (IQR)** | **Ct range** |
| --- | --- | --- |
| HPV31 | 20.0 (17.0–25.5) | 10–32 |
| HPV33 | 24.5 (19.0–28.5) | 11–33 |
| HPV35 | 20.5 (16.25–21.75) | 13–26 |
| HPV39 | 22.0 (17.0–28.0) | 9–29 |
| HPV45 | 22.0 (16.75–26.0) | 16–32 |
| HPV51 | 27.0 (17.5–29.0) | 9–31 |
| HPV52 | 22.0 (17.25–25.0) | 13–29 |
| HPV56 | 21.5 (13.75–28.5) | 11–33 |
| HPV58 | 22.0 (19.0–23.0) | 18–32 |
| HPV59 | 27.5 (19.75–31.0) | 10–33 |
| HPV66 | 23.0 (17.0–28.5) | 13–33 |
| HPV68 | 27.0 (19.0–30.5) | 8–32 |

Ct values are presented separately for each detected non-16/18 hrHPV genotype. IQR, interquartile range; hrHPV, high-risk human papillomavirus.
