# Supplementary material for: Beyond HPV16/18: genotype distribution of high-risk HPV and cycle threshold values in cervical carcinogenesis spectrum
Source: Ann Med. 2026 Oct 15;58(1):2746866. doi: 10.1080/07853890.2026.2746866 (PMC13674648; doi:10.1080/07853890.2026.2746866)
Supplement: Supplementary file 2 [file IANN_A_2746866_SM6829.docx]

**Supplementary Table S2.** Descriptive distribution of infection patterns according to histopathologic category

| **Histopathologic category** | **Single-type hrHPV (n)** | **Multiple-type hrHPV (n)** | **Total (n)** |
| --- | --- | --- | --- |
| Chronic cervicitis | 37 | 22 | 59 |
| CIN1 | 19 | 16 | 35 |
| CIN2 | 1 | 3 | 4 |
| CIN3 | **12** | **11** | 23 |
| **Total** | **69** | **52** | **121** |
