# Supplementary material for: Beyond HPV16/18: genotype distribution of high-risk HPV and cycle threshold values in cervical carcinogenesis spectrum
Source: Ann Med. 2026 Oct 15;58(1):2746866. doi: 10.1080/07853890.2026.2746866 (PMC13674648; doi:10.1080/07853890.2026.2746866)
Supplement: Supplementary file 3 [file IANN_A_2746866_SM6830.docx]

| Supplementary Table S1. Patient-level minimum Ct values by histopathologic category | | |
| --- | --- | --- |
| **Histopathology** | **Median Ct (IQR)** | **Range** |
| Chronic cervicitis | 22 (8) | 9–32 |
| CIN1 | 19 (10) | 8–32 |
| CIN2 | 17 (8) | 10–20 |
| CIN3 | 19 (7) | 11–31 |
| Ct values represent minimum patient-level Ct values. Data are presented as median (IQR) and range. | | |
